# ZNF224 enhances the oncogenic function of p21 via p53 and AKT pathways in melanoma

**DOI:** 10.1111/febs.70114

**Published:** 2025-05-05

**Authors:** Leandra Sepe, Umberto Candia, Dario Sasso del Verme, Elvira Toscano, Marianna Toriello, Gaetano Sodaro, Roberta Rapuano, Simona Romano, Michela Grosso, Giovanni Paolella, Angelo Lupo, Paola Costanzo, Elena Cesaro

**Affiliations:** ^1^ Department of Molecular Medicine and Medical Biotechnology University of Naples Federico II Naples Italy; ^2^ Ceinge Advanced Technologies Naples Italy; ^3^ Biomunex Pharmaceutical – Bioincubateur Paris Biotech Santé, Hôpital Cochin Paris France; ^4^ Department of Sciences and Technologies University of Sannio Benevento Italy

**Keywords:** AKT kinase, melanoma, p21 oncogene, p53, ZNF224

## Abstract

Expression of zinc finger protein 224 (*ZNF224*) is deregulated in various hematological and solid cancers, where its high protein levels correlate well with faster progression and worse prognosis due to activation of oncogenic pathways involved in promoting cell growth and survival, inhibiting apoptosis, and sustaining invasion and metastasis. In previous works, we identified ZNF224 as one of the mediators of the transforming growth factor beta (TGF‐β)‐induced pro‐tumoral activities in melanoma. In the present study, we thoroughly investigated the molecular mechanisms underlying the oncogenic role of ZNF224 in this kind of cancer. We demonstrated that *ZNF224* overexpression caused increased cell growth and reduced drug‐mediated apoptosis by enhancing the dysregulated function of cyclin‐dependent kinase inhibitor 1 [p21(CIP1/WAF1), also known as CDKN1A]. We provide strong evidence that *ZNF224* overexpression in melanoma cell lines positively modulated *p21(CIP1/WAF1)* gene transcription in a p53‐dependent manner and enhanced AKT‐triggered p21(CIP1/WAF1) oncogenic effects through its protein cytosolic retention, inhibiting apoptosis and favoring cell proliferation. Analysis of transcriptomic data from human melanoma tissue samples confirmed a close relationship between p21(CIP1/WAF1) and ZNF224 in cells, at least as long as p53 functionality is maintained. The tumorigenic molecular mechanism involving ZNF224, identified in this study, provides new insights into understanding melanoma development and progression, breaking ground in the research for new therapeutic tools.

## Graphical abstract

KRAB zinc finger protein ZNF224 acts as an oncogenic factor in melanoma cells, enhancing p21 cytosolic retention and impairing nuclear p21 cytostatic activity through stimulation of the AKT pathway. Furthermore, ZNF224 regulates transcriptional expression levels of p21 via p53.

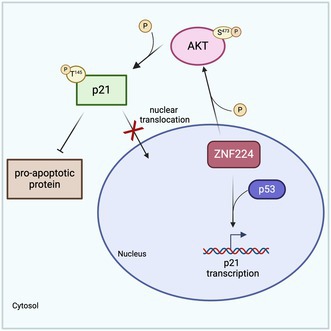

**Abbreviations**
AKTi: AKT inhibitor IV
CDKs: cyclin‐dependent kinases
CFSE: carboxyfluorescein diacetate succinimidyl ester
CIC: citrate carrier
CKIs: cyclin‐dependent kinase Inhibitors
CLL: chronic lymphocytic leukemia
CML: chronic myelogenous leukemia
FBS: fetal bovine serum
KRAB‐ZFPs: KRAB‐containing Zinc Finger Proteins
p21: p21(CIP1/WAF1)
RTqPCR: reverse transcription quantitative‐PCR
siRNA: short‐interfering RNA
WT1: Wilms tumor

## Introduction

ZNF224 is a member of one of the largest transcription factor families, KRAB‐containing Zinc Finger Proteins (KRAB‐ZFPs), originating in Vertebrates throughout evolution by gene duplications and amplifications [1, 2].

Within the KRAB‐ZFP family, ZNF224 was primarily investigated for its specific role as a transcriptional repressor of metabolic genes, such as aldolase A and mitochondrial citrate carrier (CIC) [3, 4]. Afterwards, ZNF224 showed unexpected properties of a transcriptional activator in different cellular contexts like breast cancer or chronic lymphocytic leukemia (CLL), where it causes cell proliferation and inhibits apoptosis through the transcriptional activation of *miR‐633* and *cyclin D* genes, respectively [5, 6, 7]. The regulation of its functions by a complex network of specific protein interactions is critical for the multiple and diverse activities of ZNF224 in distinctive cellular environments. In particular, the interplay between ZNF224 and its various molecular partners may contribute to the ZNF224 switch from a transcriptional repressor to a transcriptional activator and the different downstream modulation of its target genes [8, 9].

ZNF224, besides its function as a transcriptional regulator in breast cancer and CLL, acts as a cofactor of the protein of Wilms tumor (WT1) and, in such a manner, can regulate WT1 apoptotic target genes in chronic myelogenous leukemia (CML) cells to exert a tumor‐suppressive role [10, 11, 12]. Furthermore, the induction of ZNF224 expression by targeting PI3K and JAK/STAT pathways contributes to overcoming imatinib resistance in CML cells [13, 14].

In addition, ZNF224, by interacting with DEPDC1 and MED28 in bladder and breast cancer cells, respectively, stimulates two different pathways involved in the promotion and progression of tumors in these two cellular types [15, 16, 17, 18]. Therefore, it is pretty clear that ZNF224 regulates different functions in distinctive cellular milieus through a complex network of cell‐specific interactions with its molecular partners [9, 19].

Recently, we demonstrated that ZNF224 is a mediator of the pro‐oncogenic function of TGF‐β in melanoma [20], whose action is required to support tumor growth and progression, thus resulting in a strong reinforcement of cell migration and invasiveness.

The malignant transformation of melanocytes is usually characterized by uncontrolled cell division resulting from abnormalities at cell cycle checkpoints. A group of proteins, including cyclins, cyclin‐dependent kinases (CDKs), and cyclin‐dependent kinase inhibitors (CKIs), control the cell cycle of normal cells and, following DNA damage, sustain cell cycle arrest and allow DNA repair [21]. The cyclin‐dependent kinase inhibitor p21(CIP1/WAF1), a transcriptional target gene of p53, is a well‐known regulator of cell cycle progression. P21 exerts its roles as an antiproliferative effector and cell cycle inhibitor through the impairment of CDK/cyclin complexes, thus supporting the p53‐dependent cell growth arrest in G1 and S phases of the cell cycle. As a CKI, reduced p21 levels enhance tumor cell proliferation. Yet, several studies found elevated p21 levels in primary and metastatic melanomas, in which p21 acts as an anti‐apoptotic and proproliferative factor, thus promoting tumor progression [22, 23, 24, 25, 26]. More evidence indicates that p21 oncogenic functions may depend on cancer type, p21 subcellular localization, and p53 status. In particular, cytoplasmic accumulation of p21 is considered tumor‐promoting [27, 28, 29].

In this study, we strengthen our previous data on the pro‐oncogenic function of ZNF224 in melanoma [20] by demonstrating the ZNF224 involvement in cell proliferation promotion and apoptosis inhibition as a regulator of the p21 function. At first, we demonstrated that ZNF224‐induced modulation of genes associated with proliferation and apoptosis is accompanied by the concomitant induction of cell proliferation and apoptosis protection of A375 and A2058 melanoma cell lines, revealing its role as a cell proliferation promoter and apoptosis inhibitor. Interestingly, we also found that ZNF224 in A375 cells increased p21 transcription via p53. Gene expression correlation analysis performed on public datasets showed that ZNF224 expression is mostly positively correlated with p21 transcription and suggested that samples lacking this correlation are also likely to be defective in p53 activity. Finally, we found that ZNF224 overexpression promotes p21 oncogenic cytoplasmic localization through the activation of the AKT pathway, thus contributing to enhanced proliferation and survival of melanoma cells.

## Results

### ZNF224 promotes cell proliferation and protects melanoma cell lines from drug‐induced apoptosis

**Fig. 1:**
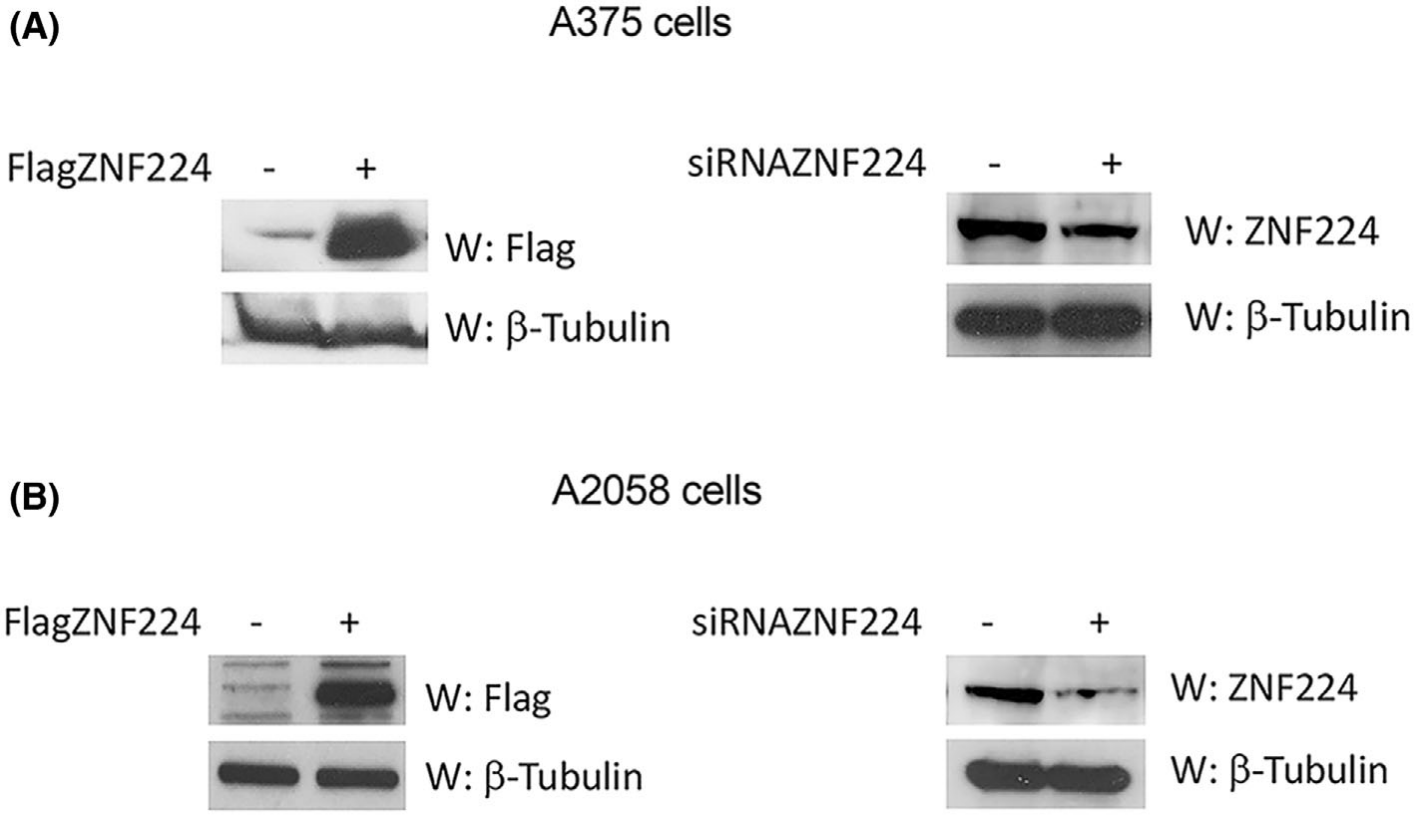
Modulation of ZNF224 expression in melanoma cell lines. ZNF224 protein levels were measured by western blot assays in A375 (A) and A2058 cells (B) transfected with p3x‐FlagZNF224 or p3x‐Flag empty vector as control by using an anti‐Flag antibody, and in cells transfected with a pool of siRNA targeting ZNF224 mRNA or a nontargeting control pool by using an anti‐ZNF224 antibody (T3). β‐Tubulin was used as a loading control.

To evaluate the pro‐proliferative and anti‐apoptotic function of ZNF224 and, consequently, investigate the molecular mechanisms involved, we overexpressed or silenced ZNF224 in A375 and A2058 melanoma cell lines (Fig. 1).

**Fig. 2:**
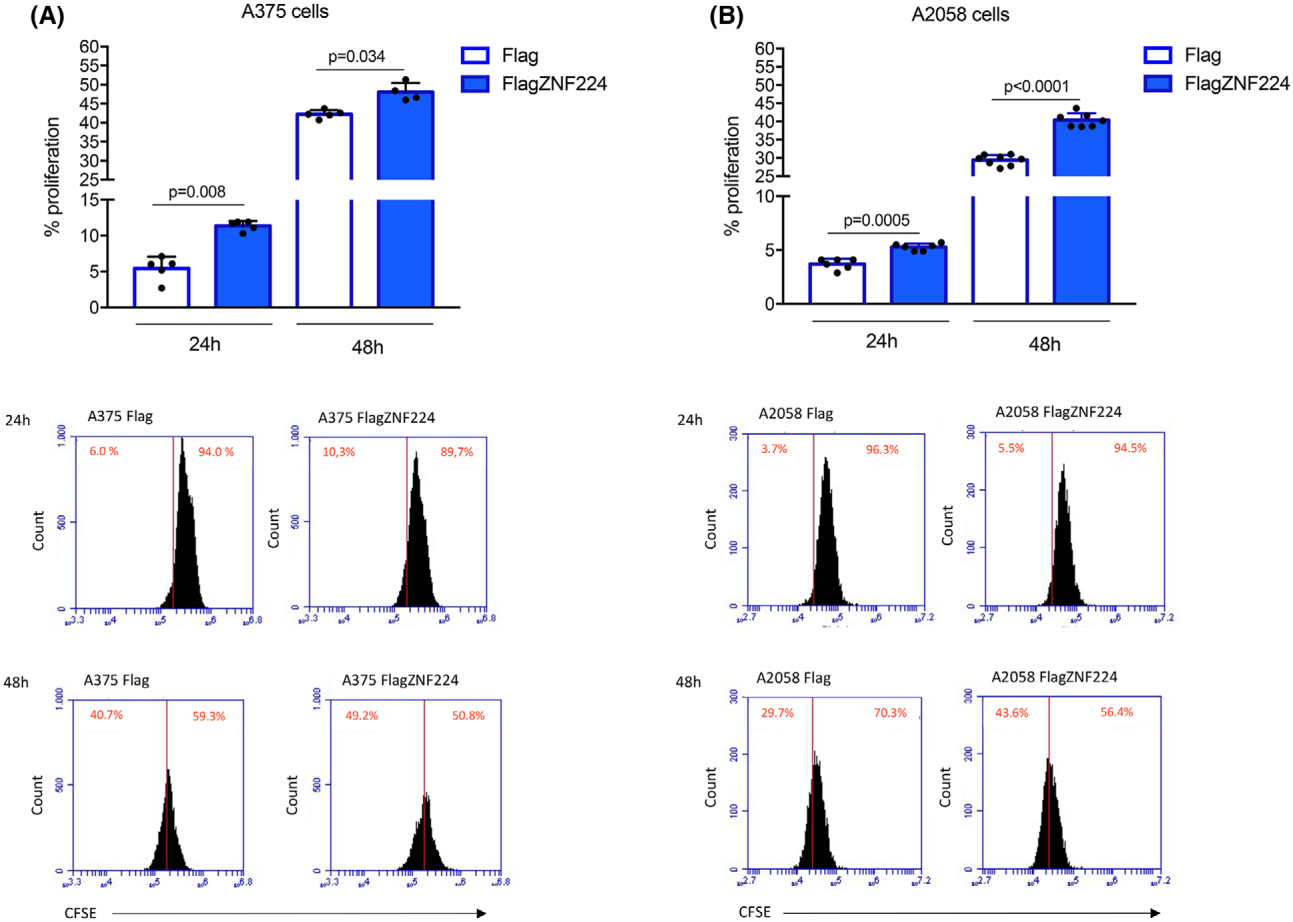
ZNF224 increases proliferation in melanoma cells. Histograms showing the relative % of proliferation in A375 (A, upper panel) and A2058 cells (B, upper panel) transfected with p3xFlagZNF224 or p3xFlag empty vector, used as control. The % of proliferation was evaluated using flow cytometry analysis of cells stained with CFSE for 24 and 48 h. The values represent the mean ± standard deviation (SD) of at least two independent experiments in duplicates. *P* ≤ 0.05 was considered a significant difference. Statistical significances were determined through the *t*‐test analysis. Representative flow cytometric analyses of A375 Flag and FlagZNF224 cells (A, lower panel), and A2058 Flag and FlagZNF224 cells (B, lower panel) 24 h and 48 h after CFSE labeling. FlagZNF224 cells exhibited a higher decrease in CFSE fluorescence intensity than the control cells (Flag). This reflects a higher dilution of CFSE and, therefore, increased cell division in FlagZNF224 cells than in control cells.

We tracked the proliferating A375 and A2058 cells overexpressing ZNF224 by flow cytometry using a carboxyfluorescein diacetate succinimidyl ester (CFSE) dye‐based assay. The measure of CFSE fluorescence intensity clearly indicated that the percentage of cells undergoing cell division was higher in ZNF224 overexpressing cells than in control cells after 24 and 48 h from CFSE staining, thus demonstrating that the overexpression of ZNF224 induced robust proliferation of melanoma cells (Fig. 2).

**Fig. 3:**
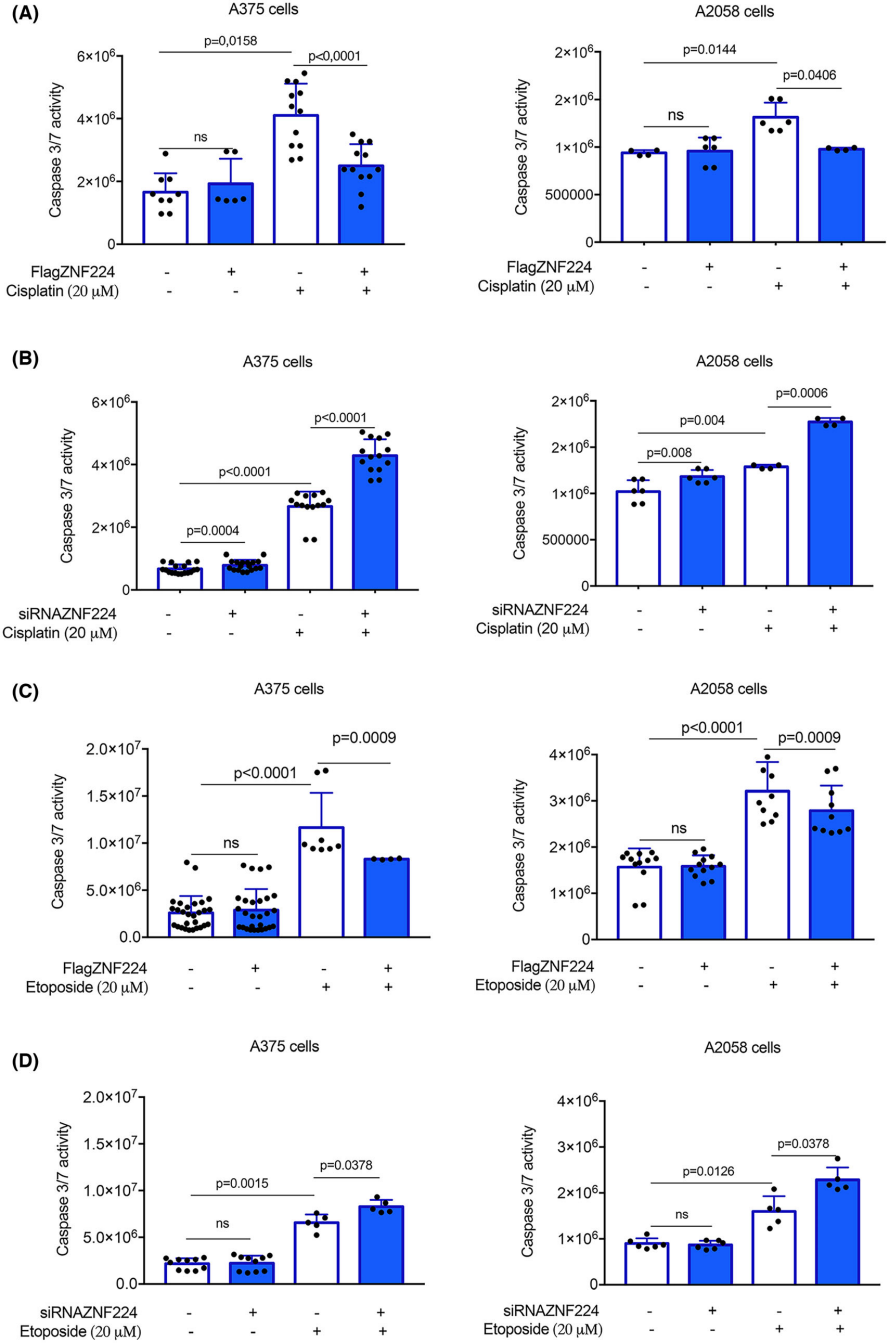
ZNF224 counteracts drug‐induced apoptosis in melanoma cells. (A) Measure of caspase‐3 and caspase‐7 activity in cisplatin‐treated A375 and A2058 cells overexpressing FlagZNF224 (+) compared to control cells (−). (B) Evaluation of caspase‐3 and caspase‐7 activity in A375 and A2058 cells silenced for ZNF224 (siRNAZNF224) (+) or not silenced (−) and treated or not with cisplatin. (C) Evaluation of caspase‐3 and caspase‐7 activity in etoposide‐treated A375 and A2058 cells overexpressing FlagZNF224 (+) compared to control cells (−). (D) Evaluation of caspase‐3 and caspase‐7 activity in A375 and A2058 silenced for ZNF224 (siRNAZNF224) (+) or not silenced (−) and treated or not with etoposide. Caspase 3/7 activity was measured using the luminescent Caspase‐Glo® 3/7 Assay. The histograms show the stable luminescent signal in response to caspase‐3/7 activities. Data shown represent the mean ± standard deviation (SD) of at least two independent experiments in duplicates. *P* ≤ 0.05 was considered a significant difference. Statistical significances were determined through the *t*‐test analysis.

Subsequently, we measured the effect of ZNF224 expression on programmed cell death of A375 and A2058 cells. To this aim, we quantified the activity of caspase 3/7 by using the Promega Caspase‐Glo 3/7 assay in conditions of ZNF224 overexpression and subsequent cisplatin treatment, a chemotherapeutic drug used for cancer therapy. These experiments demonstrated that the ectopic expression of ZNF224 significantly reduced cisplatin‐induced apoptosis through caspase 3/7 activity decrease compared to control cells (Fig. 3A). ZNF224 knockdown, on the contrary, increased the caspase 3/7 activity induced by cisplatin treatment, thus showing the protective function of ZNF224 against cisplatin‐induced apoptosis in melanoma cells (Fig. 3B). The counteracting effect of ZNF224 on drug‐induced apoptosis was also demonstrated by etoposide treatment (Fig. 3C, D).

**Fig. 4:**
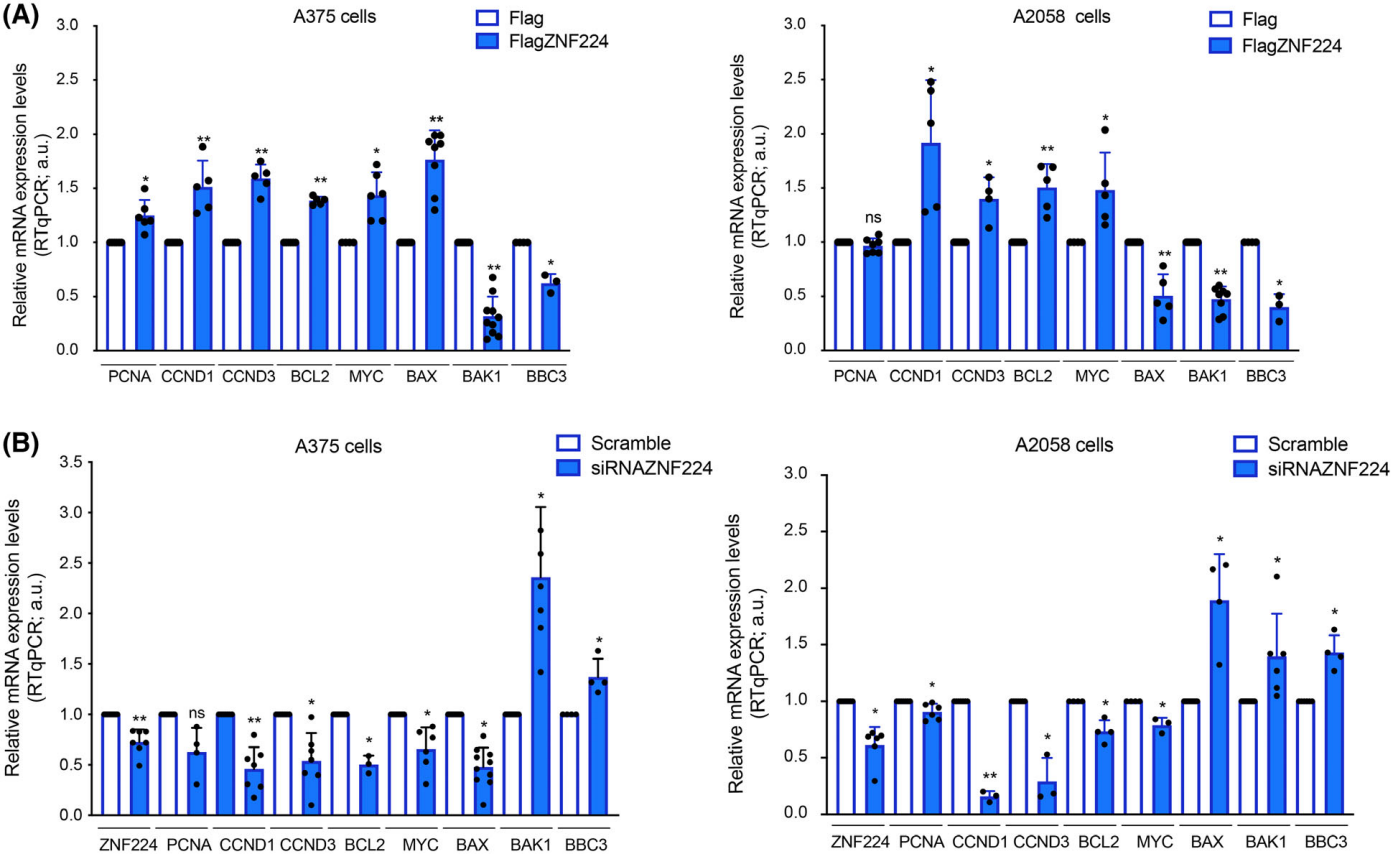
ZNF224 expression level modulation affects the expression of genes regulating apoptosis and proliferation. (A) RT‐qPCR analysis of some proliferation‐ and apoptosis‐related genes in A375 and A2058 cells overexpressing ZNF224. (B) RT‐qPCR analysis was performed in A375 and A2058 cells ZNF224‐silenced. The data shown are the mean ± SD of at least three independent experiments. *P* ≤ 0.05 was considered a significant difference (*P ≤ 0.05; **P ≤ 0.01). Statistical significances were determined through the *t*‐test analysis.

The role of ZNF224 in promoting cell proliferation and survival was supported by evaluating its ability to modulate the expression of key molecules involved in the cell cycle, proliferation, and apoptosis control. Reverse transcription quantitative PCR (RTqPCR) assays were performed on both melanoma cell lines, A375 and A2058, in which ZNF224 was overexpressed (Fig. 4A). We found that ZNF224 overexpression was accompanied by increased mRNA levels of proproliferative and anti‐apoptotic genes and decreased expression of pro‐apoptotic genes. Curiously, we observed increased expression of BAX in A375 cells overexpressing ZNF224. Literature evidence showed that in some melanoma patients, raised levels of BAX were associated with the initiation and progression of malignant melanoma [30]. As expected, the silencing of ZNF224 was associated with the down‐expression of the previously upregulated genes, thus confirming the promoting role of ZNF224 in the transcriptional modulation of these target genes (Fig. 4B).

Results of these experiments show that, in cultured cells, ZNF224 levels lead to modulation of apoptotic‐ and proliferation‐associated factors. The correlation between the expression of ZNF224 and the same genes was investigated by analyzing four transcriptomic datasets from melanoma patient samples (GSE46517, GSE19234, GSE15605, and GSE7553) selected within the NCBI Gene Expression Omnibus repository (http://www.ncbi.nlm.nih.gov/geo). In each dataset, analyzed on the Affymetrix Human Genome U133A [31] or U133 Plus 2.0 platform [32, 33], expression levels of proliferation (MYC, CCND1, CCND3, CCND2, CCNA2, CDK1 and CDK6, PCNA) and survival (BCL2, XIAP, BAX, BAK1, BBC3) regulators were compared with that of ZNF224. Bicor values obtained (see methods), used as a correlation measure, are reported in Table 1, where ZNF224 expression appears to be correlated, in the majority or in all datasets, with the expression of most genes from both proproliferative and pro‐survival gene sets with values ranging between 0.5 and 0.9.

**Table 1:** Analysis of expression levels correlation between ZNF224 and selected proliferation and survival key regulators in four human datasets from patients affected by melanoma. The correlation is expressed in terms of the bicor index (bi‐weight mid‐correlation); *P*‐value for significance is also indicated.

	GSE46517 bicor	*P*‐value	GSE19234 bicor	*P*‐value	GSE15605 bicor	*P*‐value	GSE7553 bicor	*P*‐value
XIAP	0.84	1.7E‐23	0.88	2.4E‐15	0.78	4.3E‐13	0.81	5.0E‐14
BCL2	0.83	8.2E‐22	0.88	2.2E‐15	0.66	1.8E‐08	0.79	3.7E‐13
BAX	0.63	1.8E‐10	0.36	1.5E‐02	0.64	5.2E‐08	0.61	7.5E‐07
BAK1	0.73	1.4E‐17	0.87	9.9E‐15	0.49	9.9E‐05	0.71	9.4E‐10
BBC3	0.63	1.3E‐10	0.54	1.5E‐04	0.65	4.1E‐08	0.71	9.5E‐10
CDK6	0.78	2.0E‐18	0.87	2.4E‐14	0.80	5.6E‐14	0.77	5.7E‐12
MYC	0.73	8.1E‐15	0.41	6.3E‐03	0.56	5.1E‐06	0.34	1.1E‐02
CCNA2	0.70	1.9E‐13	0.76	2.3E‐09	0.56	5.6E‐06	0.47	2.5E‐04
CCND3	0.69	6.5E‐13	0.87	2.7E‐14	0.63	9.3E‐08	0.85	5.5E‐17
CCND2	0.67	5.8E‐12	0.81	1.8E‐11	0.53	2.3E‐05	0.85	1.7E‐16
CDK1	0.62	3.6E‐10	0.61	1.1E‐05	0.57	2.6E‐06	0.40	2.2E‐03
PCNA	0.60	2.1E‐09	0.52	3.3E‐04	0.62	2.2E‐07	0.32	1.7E‐02
CCND1	0.34	1.5E‐03	0.67	7.8E‐07	0.51	3.9E‐05	0.19	1.6E‐01

### ZNF224 overexpression increases the p53‐dependent transcriptional activation of p21

**Fig. 5:**
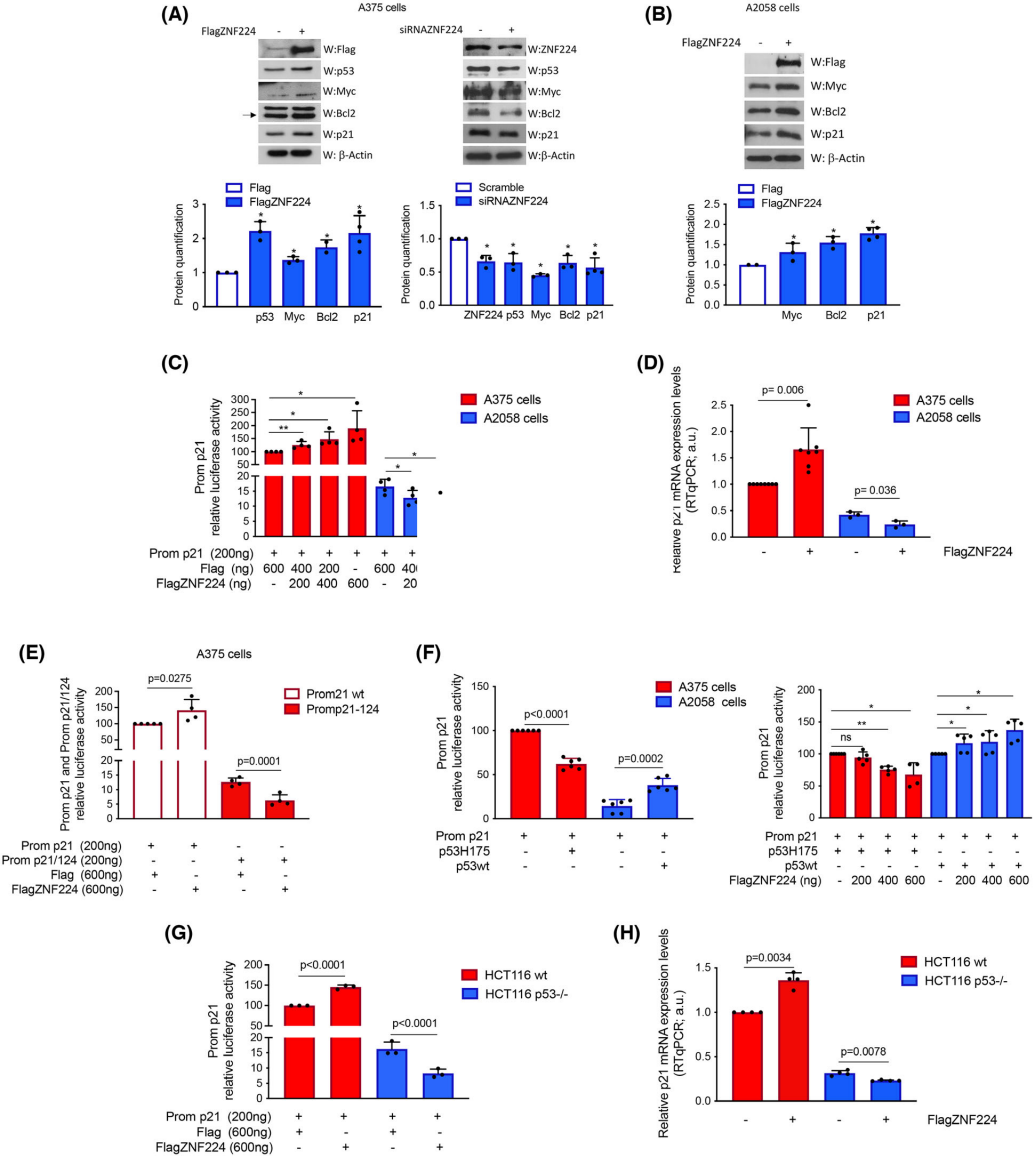
ZNF224 modulates p21 gene expression in melanoma and colon cancer cell lines. (A) Analysis by western blotting assay of the expression levels of markers of cell growth and apoptosis in A375 melanoma cells overexpressing ZNF224 (left panel) or ZNF224‐silenced (right panel), compared to cells transfected with the empty vector Flag (−) or a scramble siRNA (−), respectively. The arrow indicates the specific band. (B) Analysis by western blotting assay of c‐Myc, Bcl2, and p21 in A2058 melanoma cells overexpressing ZNF224. β‐Actin was used as a loading control. Protein quantification was performed by using ImageJ software. (C) Transcriptional activity of the p21 promoter (Prom p21) was measured by luciferase reporter activity in A375 and A2058 cells transfected with increasing amounts of p3xFlagZNF224 or p3xFlag vectors. (D) Expression levels of p21 mRNA were evaluated by RTqPCR in A375 and A2058 cells overexpressing ZNF224. (E) Luciferase reporter activities of Prom p21 and Prom p21/124 in A375 cells transfected with p3xFlagZNF224 and/or p3xFlag vectors. (F) Luciferase assay was performed on Prom p21 cotransfected with the p53H175 plasmid, encoding for the mutant p53 protein, in A375 cells or with the p53 wt plasmid, encoding for wild‐type p53, in A2058 cells (left panel). Luciferase assay was performed on Prom p21 cotransfected with the p3xFlagZNF224 plasmid and p53H175 in A375 or p53 wt in A2058 cells (right panel). In all luciferase assays, the Renilla luciferase activity was measured to normalize the promoter activity. Data shown are the means ± SD of at least three independent experiments. (G) Luciferase reporter activity of Prom p21 was evaluated in HCT116 wt and HCT116 p53−/− cells transfected with FlagZNF224 or Flag vectors. (H) Expression levels of p21 mRNA were evaluated by RTqPCR in HCT116 wt and HCT116 p53−/− cells overexpressing ZNF224. The data shown are the mean ± SD of at least three independent experiments. *P* ≤ 0.05 was considered a significant difference (*P ≤ 0.05; **P ≤ 0.01). Statistical significances were determined through the *t*‐test analysis.

To delve deeper into the molecular mechanisms by which ZNF224 could be involved in the proliferation and survival of melanoma cells, we checked at the protein level whether typical regulators of the cell growth and apoptosis were affected by ZNF224 modulation. The results in Fig. 5A show that ZNF224 overexpression in A375 cells was accompanied by an increase in p53 and a group of p53‐regulated factors, including p21, the oncogene c‐Myc, and Bcl2. Conversely, decreased expression of these proteins was observed in cells silenced for ZNF224 by short‐interfering RNA (siRNA)‐mediated knockdown. In addition, in A2058 cells the c‐Myc, Bcl2, and p21 proteins increased following FlagZNF224 overexpression (Fig. 5B).

The upregulation of p53 and p21 by ZNF224 was unexpected. In this study, we focused our attention on p21, a critical molecular player involved in p53‐mediated cell cycle checkpoints already proven to have a dual and divergent role in cancer progression. To examine the molecular mechanism by which ZNF224 acts on p21 transcription, we performed a luciferase reporter assay both in A375 cells, which bear wild‐type p53 gene status, and in A2058 cells, which show a mutation in the DNA‐binding domain of p53. A plasmid containing the promoter region of p21 cloned upstream of the luciferase reporter gene (Prom p21) was cotransfected with the expression vector for ZNF224 (p3x‐FlagZNF224) and/or an empty control vector (p3x‐Flag) and luciferase activity was evaluated. We found that ZNF224 overexpression induced a dose‐dependent increase in Prom p21 transcriptional activity in the A375 melanoma cell line. In contrast, it repressed Prom p21 in the A2058 cell line (Fig. 5C). To reinforce these data pointing out the regulatory function of ZNF224 on the transcription of p21, RTqPCR assays were performed in A375 cells. Hence, we demonstrated that ZNF224 overexpression is accompanied by increased levels of p21 mRNA in A375 cells, whereas it produced a reduction in p21 mRNA amount in A2058 cells (Fig. 5D). These data indicated that ZNF224 differentially influenced the p21 promoter activity and p21 mRNA amount depending on wild‐type or mutated p53 protein.

To confirm our results, we also performed luciferase assays in A375 cells using the p21/124 construct (Prom p21/124), which suffered a large deletion, leading to the loss of two p53 DNA‐binding sites in the p21 promoter [34]. As shown in Fig. 5E, the overexpression of ZNF224 increased the transcriptional activity of Prom p21, as expected, while it reduced that of Prom p21/124, which lacks p53 binding sites.

To ultimately validate that increased p21 gene transcription mediated by ZNF224 is dependent on wild‐type p53, we first performed the luciferase assays in A375 cells co‐transfected with Prom p21 and the p53H175 plasmid, encoding a p53 mutant lacking DNA‐binding activity [35], and, in A2058 co‐transfected with Prom p21 and the p53 wild‐type plasmid. This experiment demonstrated that the presence of functional wild‐type p53 was required to induce a significant luciferase activity of Prom p21. Overexpression in A375 of the p53 mutant isoform (p53H175), indeed, produced a diminished transcriptional activity of Prom p21, likely competing with wild‐type endogenous p53, whereas the overexpression of wild‐type p53 in A2058 was able to promote a clearly p53‐mediated transcription of Prom p21 (Fig. 5F, left panel). Subsequently, we demonstrated that FlagZNF224, when overexpressed with p53H175 mutant, further decreased the Prom p21 activity in A375 cells. Conversely, in A2058 cells, FlagZNF224, when co‐expressed with p53 wild‐type plasmid, increased the Prom p21 activity (Fig. 5F, right panel).

We finally tested the observed effect of ZNF224 overexpression on p21 expression in HCT116 colorectal cancer cell lines, p53 wild‐type (HCT116 p53+/+) or p53 null (HCT116 p53−/−). As already demonstrated in melanoma cells, we found an enhanced p21 promoter luciferase activity in HCT116 p53+/+ and a reduction in HCT116 p53−/− cells (Fig. 5G). Coherently, p21 mRNA levels increased in HCT116 p53+/+ and decreased in HCT116 p53−/− (Fig. 5H). These results demonstrate that p21 transcriptional activation mediated by ZNF224 strictly depends on the presence of wild‐type p53. Interestingly, in A2058 cells, we also observed that the positive regulation of p21 protein following FlagZNF224 overexpression did not correlate with decreased p21 mRNA levels. Therefore, these results suggest that ZNF224 may be involved in different mechanisms that regulate p21 levels post‐transcriptionally.

### ZNF224 influence on p21 expression occurs in human melanoma samples and depends on fully active p53

The observation that ZNF224 overexpression enhanced p21 expression in melanoma cell lines prompted us to confirm a similar role in human melanoma biopsies. The possibility of coordinated expression of ZNF224 and p21 was tested by evaluating the correlation between their expression levels in the above‐described transcriptomic datasets: GSE46517 and GSE19234 (Table 2) confirms the existence of such a correlation with bicor values of 0.65 and 0.60, respectively, and small *P*‐values; a weaker correlation was observed in the other two datasets, GSE7553 and GSE15605, where bicor values do not go beyond 0.36 and 0.24, and the larger *P*‐values fail to support it, at least with the number of subjects involved. These lower bicor values do not appear to depend on a reduced ability of ZNF224 to affect p53 levels, as the expression levels of the two genes are strongly correlated in all datasets, with bicor values ranging from 0.56 to 0.70 (Table 2). This correlation is also consistent with the effects of ZNF224 on p53 levels observed in cultured melanoma cells. The weaker correlation between ZNF224 and p21 observed for GSE7353 and GSE15605 raises the possibility that in these datasets p53 functionality might be completely, or at least partially, lost as a result of somatic mutations or other mechanisms. This interpretation would be supported by the fact that when testing the correlation between p53 and a panel of known p53 target genes [35], 47 and 43 out of 59 tested genes showed bicor values (Table S1; Fig. S1) lower than in GSE46517 used as a reference.

**Table 2:** The analysis of correlation of ZNF224 expression levels with p21 and with p53 and of p53/p21 in four human datasets from patients affected by melanoma. The correlation is expressed in terms of the bicor index (bi‐weight mid‐correlation); *P*‐values for significance is also indicated.

Dataset	ZNF224/P21 bicor	*P*‐value	ZNF224/P53 bicor	*P*‐value	P53/P21 bicor	*P*‐value
GSE46517	0.65	4.4E‐11	0.70	2.3E‐13	0.69	5.2E‐13
GSE19234	0.60	1.9E‐05	0.56	9.1E‐05	0.46	1.5E‐03
GSE7553	0.36	5.8E‐03	0.62	3.6E‐07	0.35	8.4E‐03
GSE15605	0.24	7.4E‐02	0.57	2.8E‐06	0.46	3.0E‐04
GSE7553 (p53wt‐like)	0.48	4.8E‐04	0.58	1.5E‐05	0.58	1.2E‐05
GSE15605 (p53wt‐like)	0.07	6.3E‐01	0.40	6.5E‐03	0.63	4.7E‐06

**Fig. 6:**
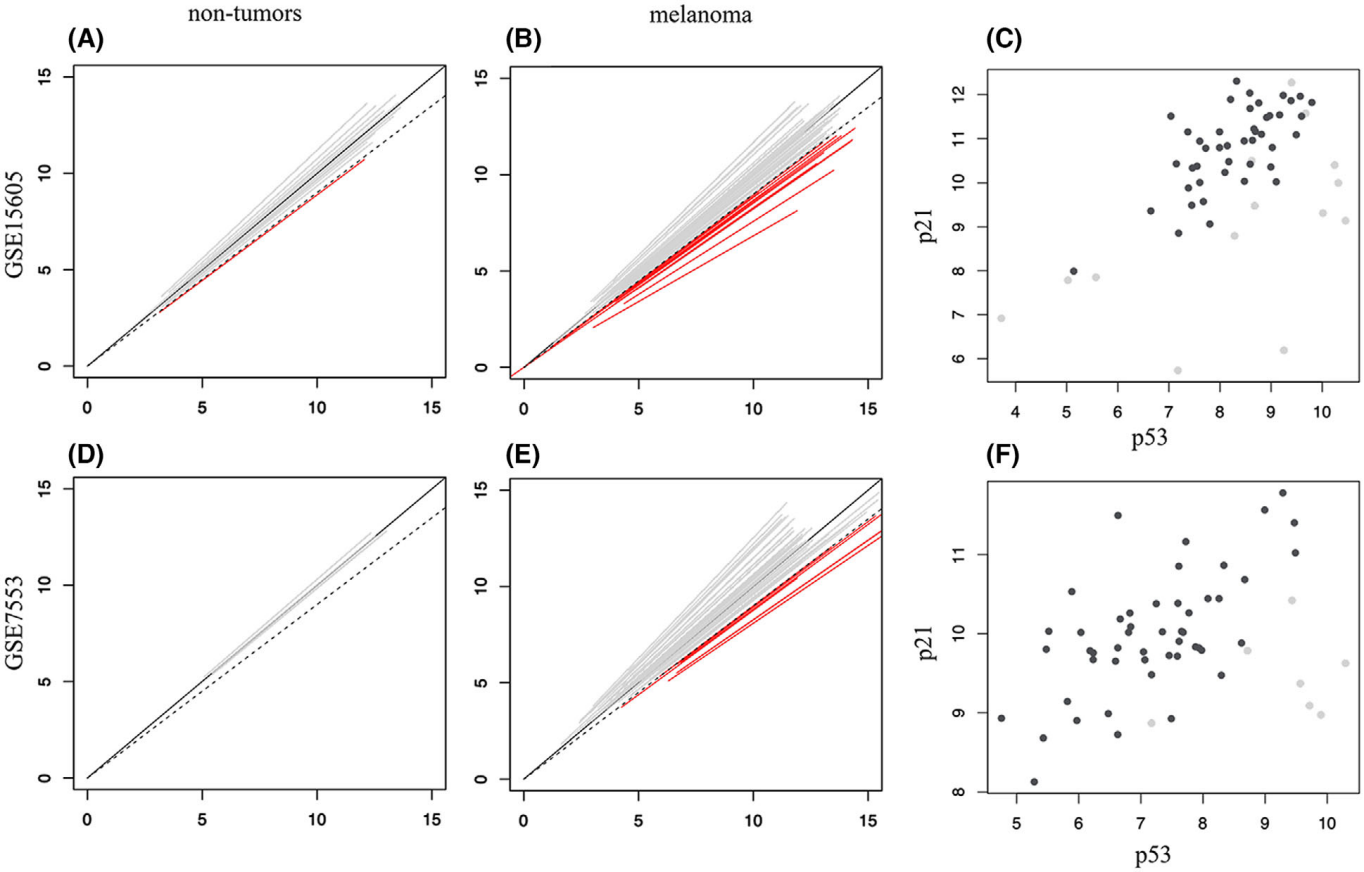
Inferring p53 effectiveness in regulating the expression level of its target genes. In panels (A), (B), (D), and (E) straight lines represent, for individual samples from GSE15605 and GSE7553 datasets, the expression profile of 59 genes transcriptionally regulated by p53 and correspond to the best fitting segment calculated for observed vs expected values for each gene. (A) and (D) correspond to nontumor, while (B) and (E) to melanoma samples: lines with slope between 1.1 and 0.9 are colored in gray and identify ‘normal‐like’ samples with observed expression profiles very similar to the expected ones; red lines with slope values below the limit of 0.9 (indicated with a dashed gray line), refer to ‘p53‐impaired’ samples with p53 target genes expressed lower than expected. (C, F) The expression levels of p21 are reported as a function of p53 for normal‐like samples (dark gray dots) as well as for p53‐impaired ones (light gray dots) for GSE15605 (C) and GSE7553 (F).

Considering these results, the weak ZNF224/p21 correlation observed in some melanoma datasets might still be due to the presence of a fraction of samples with impaired p53 function. To test this hypothesis, a procedure was set up aimed to test p53 ability to influence the overall expression of its target genes by comparing their transcript levels with levels observed for the same genes in nontumor samples of the same dataset. Briefly, the procedure builds on the idea that most cells from nontumor tissue samples have normally functioning p53: these samples were used to relate expression levels of p53 with those of a panel of 59 genes, known to be transcriptionally regulated by p53 [35]. For each gene, this relationship was evaluated by curve fitting analysis to determine the parameters of the resulting function. For each melanoma sample, an ‘expected’ level was estimated for each p53 target, by using the previously calculated parameters, and compared with the measured ones. The straight line describing this measured vs expected relationship was determined, again by curve fitting analysis, and samples with a slope between 1.1 and 0.9 were considered ‘normal‐like’, that is, with fully functioning p53, while samples with lower slope values were assumed to have impaired functionality of p53 itself or of other factors acting downstream of it. The procedure showed that, in datasets GSE7553 and GSE15605, melanoma samples (Fig. 6B,E) have greater variability than nontumor ones (Fig. 6A,D), as they include a fraction (13% and 24%) of samples with impaired response to p53, as indicated by their reduced line slope. Interestingly, these samples, reported as gray dots in panels C and F of Fig. 6, are mostly located in the lower‐right part of the graph, away from the majority of the other samples and potentially reducing the quality of the overall p53/p21 correlation. Removal of these p53‐impaired samples, followed by recalculation of p53/p21 correlation, produced values (see normal‐like datasets in Table 2) much higher than those obtained before, with bicor values increased from 0.35 to 0.58 and from 0.46 to 0.63. This result confirms the expected p53 action on p21 expression in melanoma samples, even in datasets where an apparently weaker relation was measured due to the presence of a fraction of samples with impaired p53 functionality. Except for the GSE15605 dataset where ZNF224/p21 correlation is not numerically trustworthy (*P*‐value extremely high), the bicor values for ZNF224/p21 after removal of the p53‐impaired samples showed a marked increase (Table 2), with three of the four datasets exhibiting a clear correlation between ZNF224 and p21 expression levels. These results corroborate the notion that ZNF224 is involved in the regulation of elevated p21 transcript levels in melanoma patient samples, as it is in melanoma cell lines, and that this effect is reliant on a p53‐dependent mechanism.

### ZNF224 influences the subcellular localization of p21

The above‐described proproliferative and anti‐apoptotic function of ZNF224 in melanoma cell lines prompted us to investigate whether the increased p21 expression, observed in A375 cells, could be a suitable cause of ZNF224‐induced proliferation, which leads to the dramatic increase of melanoma cell growth, rather than to cell cycle arrest and induction of apoptotic events.

**Fig. 7:**
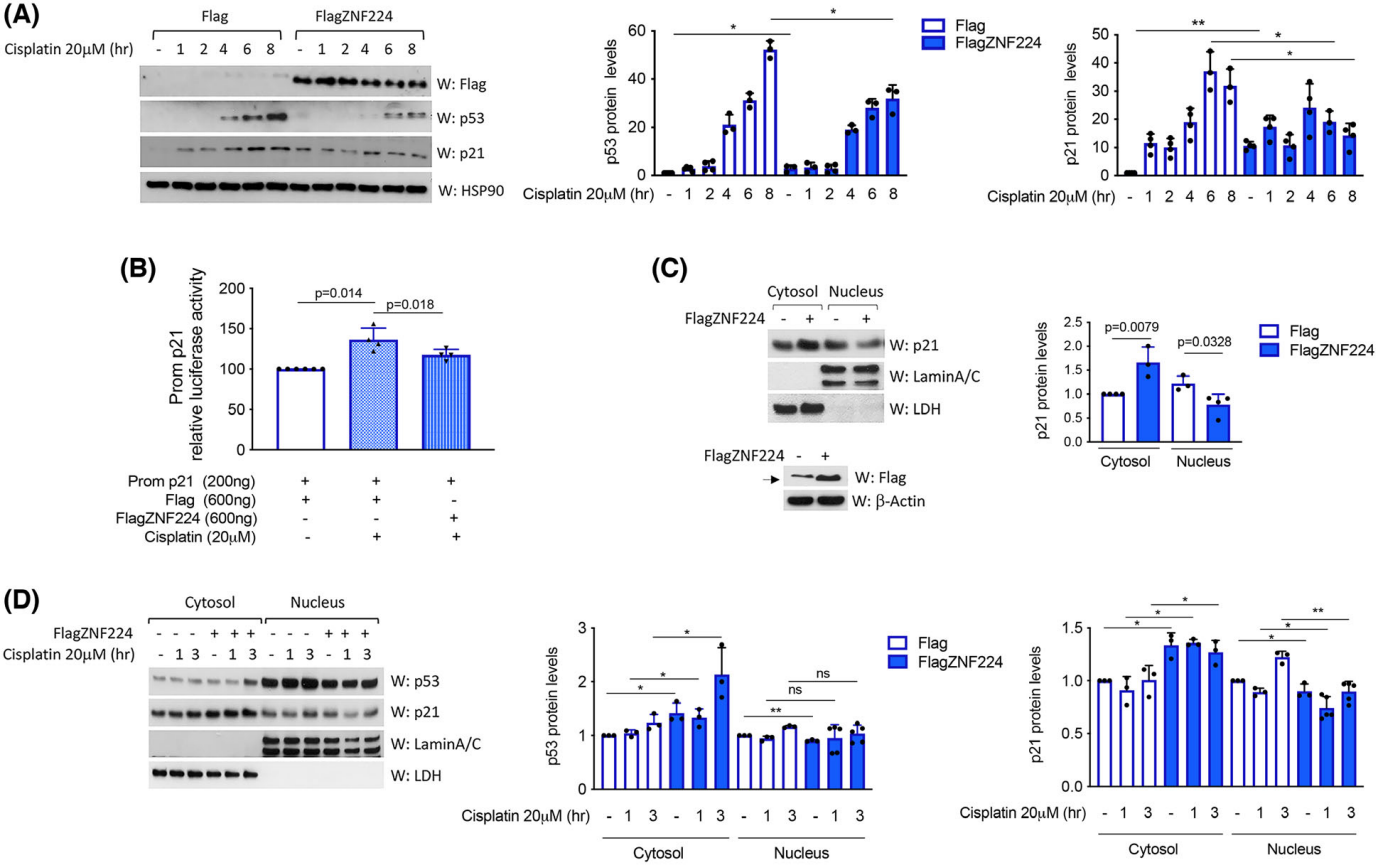
ZNF224 counteracts the cisplatin‐mediated induction of p21 and increases cytosolic p21 levels. (A) Western blot assay of p53 and p21 expression in control A375 cells (Flag) or overexpressing ZNF224 (FlagZNF224) and treated with cisplatin at various times. (B) Luciferase activity of Prom p21 in A375 cells transfected with p3xFlagZNF224 or p3xFlag as control and treated with cisplatin or not. (C) Western blot assay of p21 expression in cytoplasmic and nuclear fractions of A375 cells overexpressing FlagZNF224 (top panel). The expression of FlagZNF224 was controlled by hybridization with the anti‐Flag antibody (bottom panel). The arrow indicates the specific band (D) Western blot assay of p53 and p21 expression in cytoplasmic and nuclear fractions of A375 cells overexpressing ZNF224 and treated with cisplatin at different times. LaminA/C and LDH antibodies were used to control nuclear and cytosolic fraction purity, respectively. Protein quantification was performed by using imagej software (right panels of Fig. 7A,C,D). All data were assessed as the means ± standard deviation (SD) of at least three independent experiments. *P* ≤ 0.05 was considered a significant difference (*P ≤ 0.05; **P ≤ 0.01). Statistical significances were determined through the *t*‐test analysis.

To address this issue, we first evaluated, by western blot assay, the effects of ZNF224 overexpression in modulating p21 expression following cisplatin treatment. Results shown in Fig. 7A indicated that cisplatin treatment increased p53 and p21 protein levels in control A375 cells, according to literature data indicating that one of the mechanisms of cisplatin‐induced apoptosis in melanoma cells is p53‐mediated [36]. However, we found that high expression of ZNF224 counteracted the cisplatin‐induced p53 and p21 expression while causing the increased p53 and p21 protein levels in the absence of drug treatment. Furthermore, luciferase assay, which was carried out after transient transfection of the expression vector for ZNF224, showed that ZNF224 hampered the activation of the p21 promoter induced by cisplatin treatment (Fig. 7B). These findings indicate that ZNF224 exerts a negative control at the transcriptional level on the p53/p21 pathway in the presence of an apoptotic stimulus.

The presence of p21 in the cell nucleus is associated with the arrest of the cell cycle progression. In contrast, when its localization is restricted to the cytoplasm, p21 works as a proproliferative and anti‐apoptotic factor [27, 28, 29]. Therefore, we wondered whether ZNF224 overexpression also modulated the p53/p21 pathway in A375 cells by affecting the distribution of p21 in the intracellular compartments and consequently its role. To this aim, we overexpressed ZNF224 and analyzed p21 subcellular distribution by western blot analysis. Interestingly, a significant increase in cytosolic p21 and a concomitant reduction of nuclear p21 in ZNF224‐overexpressing cells were observed (Fig. 7C).

Data from other laboratories indicated that cytosolic p21 localization contributes to chemoresistance in many cancer types [37, 38]. Following this critical evidence, we demonstrated in Fig. 7D that ZNF224 overexpression caused an increase in p21 cytosolic levels after cisplatin treatment, thus suggesting that ZNF224 exerted a protective effect toward cisplatin‐induced apoptosis through an increased p21 cytoplasmic localization.

### ZNF224 promotes AKT‐mediated phosphorylation of p21 and pushes toward proliferation in AKT‐dependent manner

**Fig. 8:**
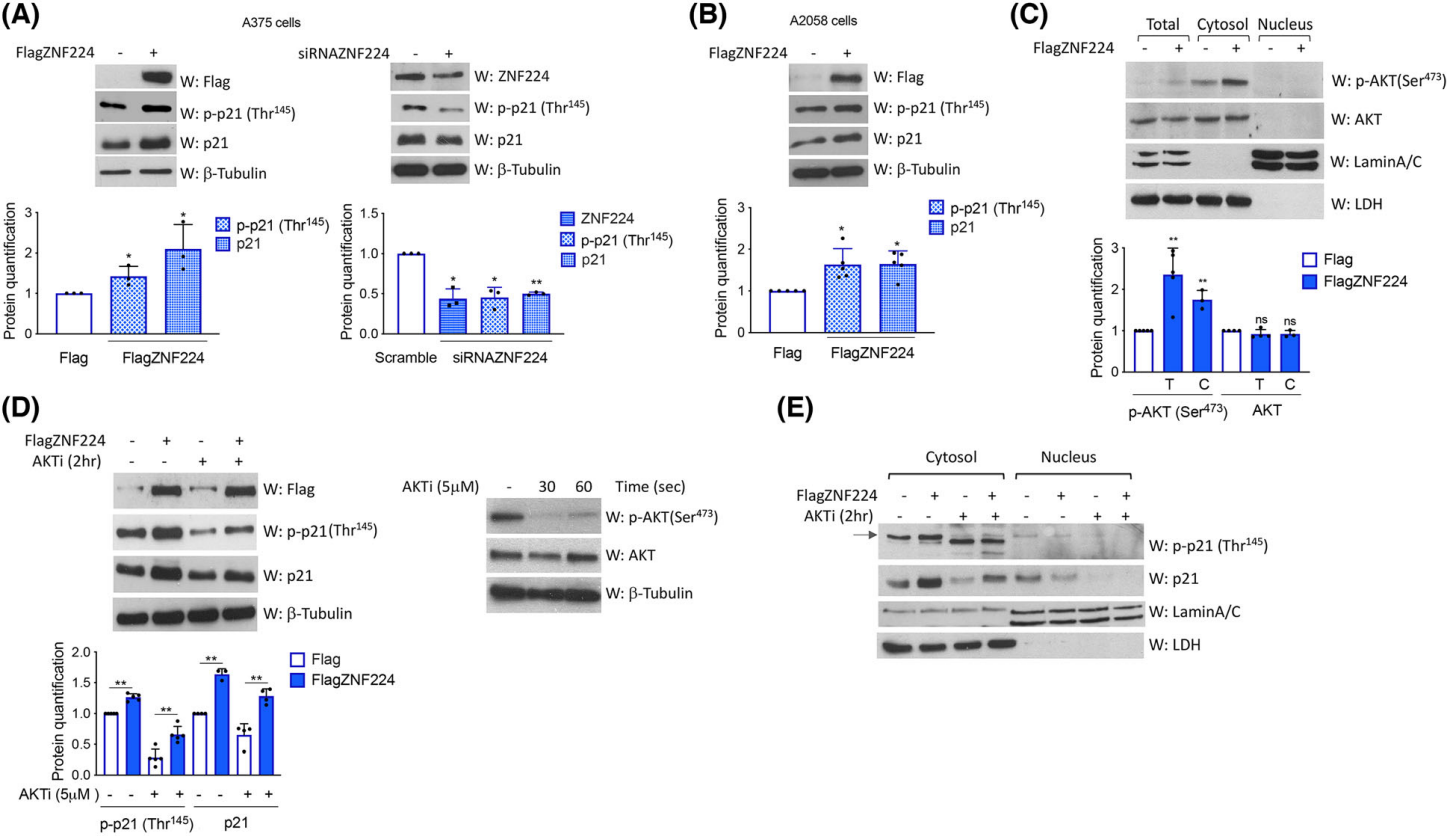
ZNF224 induces the AKT‐mediated p21 phosphorylation. (A) Western blot assay of p21 and p‐p21 Thr^145^ levels in A375 cells transfected with p3xFlag ZNF224 (+) or empty vector p3xFlag (−) (left panel). The right panel shows a western blot assay of p21 and p‐p21 Thr^145^ levels in A375 cells transfected with ZNF224‐specific siRNAs (+) or scramble siRNAs (−). The levels of β‐tubulin were measured as a loading control. (B) Western blot assay of p21 and p‐p21 Thr^145^ levels in A2058 cells transfected with p3xFlagZNF224 (+) or empty vector p3xFlag (−) (C) Western blot assay of AKT and p‐AKT Ser^473^ expression in total, cytoplasmic, and nuclear protein extracts from A375 cells transfected with p3xFlagZNF224 (+) or empty vector p3xFlag (−). The level of LDH and lamin A/C was used to control nuclear and cytosolic fraction purity, respectively. (D) Western blot assay of p‐p21 Thr^145^ and p21 levels in A375 cells transfected with p3xFlagZNF224 or empty vector p3xFlag and treated or not with AKT inhibitor IV (AKTi). The efficacy of treatment with AKTi was checked at 30 and 60 s in A375 cells, evaluating the reduction of p‐AKT Ser^473^ expression (right panel). The levels of β‐tubulin were measured as a loading control. (E) Western blot assay showing the cytoplasmic/nuclear localization of p21 and p‐Thr^145^ p21 in A375 overexpressing FlagZNF224 compared to the control cells with or without AKTi treatment. The arrow indicates the specific band. In all experiments, protein quantification was performed by using imagej software based on densitometric analysis. Data shown are the means ± standard deviation (SD) of at least three independent experiments. *P* ≤ 0.05 was considered a significant difference (*P ≤ 0.05; **P ≤ 0.01). Statistical significances were determined through the t‐test analysis.

The p21 protein structure is characterized by the presence of some phosphorylation sites targeted by different kinases. Among them, the protein kinase B/AKT, a survival kinase usually hyperactivated in many tumors [39], is responsible for p21 phosphorylation at the Ser^146^ residue. This event significantly increases p21 protein stability, while an additional phosphorylation at the Thr^145^ residue mediated by the protein kinase B/AKT induces p21 cytoplasmic accumulation [40]. Hence, to investigate whether the ZNF224‐dependent increase in cytosolic p21 was due to increased phosphorylation levels, we measured phosphorylated and total p21 protein by western blot analysis in A375 cells. Remarkably, we found that ZNF224 overexpression was accompanied by an induction of p‐Thr^145^ p21 and an increase in total p21. The latter result was, at least partially, due to the previously described p53‐mediated effect of overexpressed ZNF224 on p21 transcription. Accordingly, we observed reduced expression levels of both p21 and p‐Thr^145^ p21 in A375 cells ZNF224‐silenced (Fig. 8A). Also, we showed that FlagZNF224 overexpression increased p‐Thr^145^ p21 in A2058 cells, bearing PTEN deletion that results in constitutive high PI3K/AKT pathway activity (Fig. 8B) [41].

Altogether, these results indicate that ZNF224 could influence p21 post‐translational modification, most likely related to AKT activation.

Thus, we decided to verify whether the phosphorylation of p21 on Thr^145^ by ZNF224 depends on AKT. At first, we evaluated the level of phosphorylated AKT on Ser^473^, a typical modification for AKT activation [39], in A375 cells overexpressing ZNF224. As shown in Fig. 8C, we observed that the ectopic expression of ZNF224 in A375 cells was accompanied by increased p‐Ser^473^ AKT levels with respect to control cells, already visible in the total extract but more evident in the cytosolic fraction. The expression levels of total AKT protein were unchanged.

To demonstrate whether AKT activation was necessary for p21 phosphorylation mediated by ZNF224 overexpression, we treated A375 cells overexpressing ZNF224 with AKT inhibitor IV (AKTi), which specifically blocks AKT phosphorylation/activation, and then performed western blot analysis. As shown in Fig. 8D, the treatment with AKTi counteracted the p21 phosphorylation induced by ZNF224, thus demonstrating that active AKT is required for ZNF224‐induced p21 phosphorylation. Besides, AKT inhibition also reduced total p21 protein levels, likely due to decreased p21 protein stability [38] (Fig. 8D). These results indicate that ZNF224 may act as a positive regulatory component of the AKT/p21 pathway, being involved in the activation of AKT and subsequent p21 phosphorylation. Furthermore, fractionated protein extracts showed that cytoplasmic p‐Thr^145^ p21 was affected by ZNF224 overexpression and AKTi treatment (Fig. 8E).

**Fig. 9:**
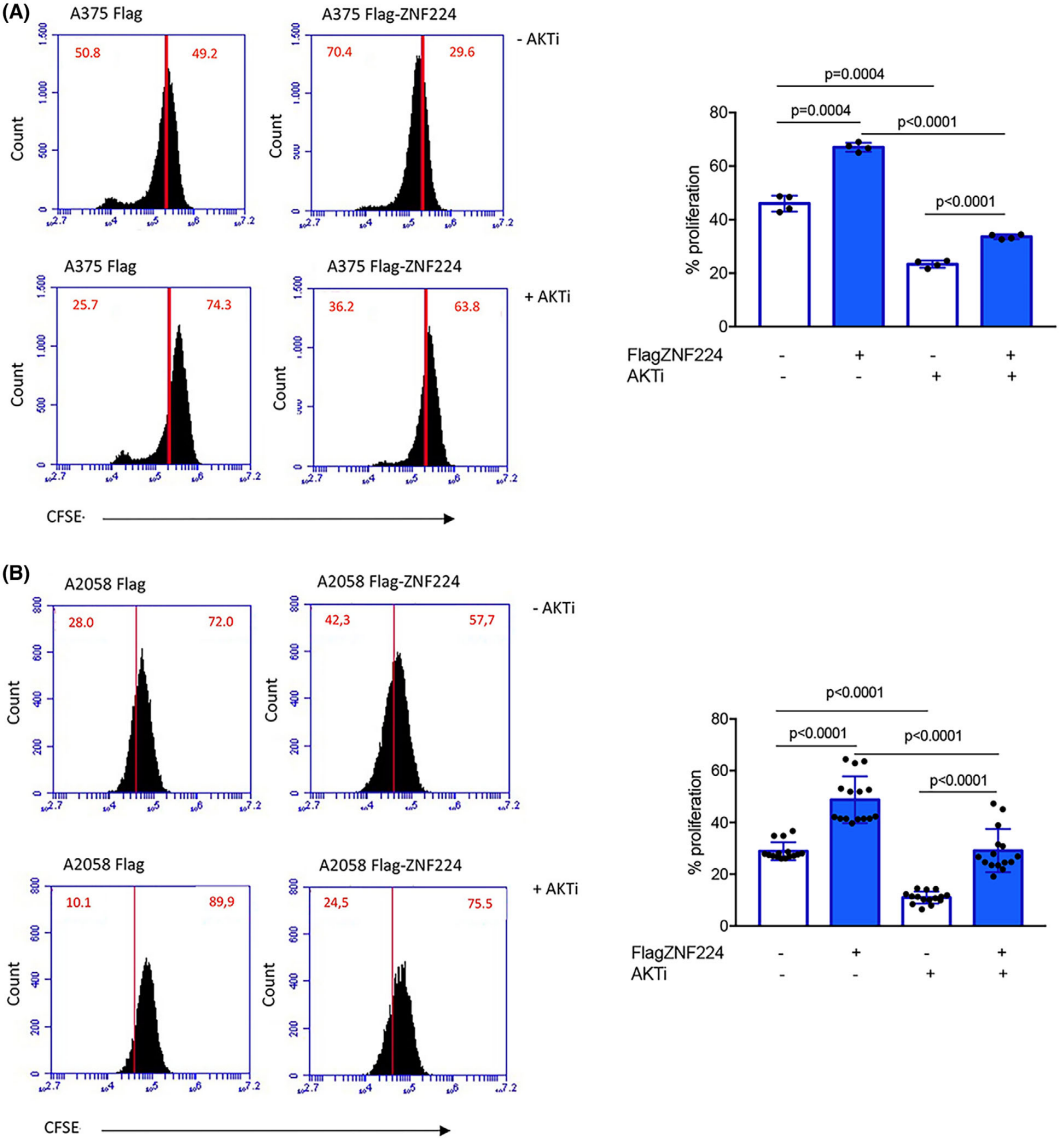
The AKT inhibition counteracts the proliferative effect of ZNF224. Flow cytometric analysis of CFSE‐labeled melanoma cells overexpressing ZNF224 in the presence of AKT inhibitor IV (AKTi). A375 (A) and A2058 (B) cells transfected with the p3xFlagZNF224 plasmid or p3xFlag empty vector were labeled with CFSE and then treated with the AKTi for 16 h. The percentage of dead cells excluded from the analysis was determined by 7AAD labeling. Histograms show the % of proliferation and were obtained from the means ± standard deviation (SD) of at least two independent experiments in duplicates. *P* ≤ 0.05 was considered a significant difference. Statistical significances were determined through the ANOVA test.

Finally, we evaluated whether the ability of ZNF224 to induce cell proliferation in melanoma cell lines was dependent, at least in part, on its role in the activation of AKT. To assess the role of AKT activation on ZNF224‐induced proliferation, we conducted CFSE cell proliferation assays in the presence of the AKT inhibitor IV in A375 cells overexpressing ZNF224. Figure 9A shows that the treatment with AKT inhibitor IV impaired the proliferation of A375 control cells and significantly counteracted the proproliferative effect induced by ZNF224. The CFSE cell proliferation assay was also performed in A2058 cells following ZNF224 overexpression and AKT inhibitor IV treatment (Fig. 9B). As well as in A375, AKT inhibition reduced the proliferative function of ZNF224, thus suggesting that activated AKT is crucial for ZNF224‐mediated proliferation modulation in both melanoma cell lines. Overall, these results show the influence of ZNF224 levels on AKT‐dependent p21 phosphorylation, which may be a determinant of ZNF224's ability to induce proliferation.

## Discussion

The balance between cell proliferation and cell death constitutes an essential feature of living organisms that need to control cellular homeostasis during their physiological processes as differentiation and development. Several functional proteins are meant to control the numerous steps along the differential pathways, and dysregulation of the fine balance between proliferative and apoptotic stimuli strongly drives carcinogenesis. Different factors have been proposed as guardians of the interplay between these stimuli. Therefore, their amplification, overexpression, or loss may finally cause constitutive activation of an oncogenic pathway, thus driving the initiation and progression of cancer.

The transcription factor ZNF224 has been shown to be involved in numerous pathways related to cell survival, cell death, and cell cycle in various human cancers through its interaction with different molecular partners. The pathogenic role of ZNF224 is cancer‐type specific and tumor context‐dependent, being able to act as an oncogene or a tumor suppressor in human cancers [9, 19]. Recently, we reported that ZNF224 expression supports the constitutive activation of the pro‐oncogenic pathway of TGF‐β in melanoma, through the upregulation of TGF‐β itself, its receptors, and various TGF‐β target genes involved in epithelial‐mesenchymal transition [20].

Here, we identify an additional mechanism used by ZNF224 to affect melanoma progression. Indeed, we demonstrated that sustaining the oncogenic function of the p21 protein is one of the ways by which ZNF224 promotes strong melanoma cell proliferation and inhibits cell death. In this context, it is to be noted that p21, whose expression is induced by TGF‐β, plays a role in the transcriptional activation of several TGF‐β pro‐metastatic and pro‐invasive target genes in some cancer cells [42]. The function of p21 is mainly dependent on its expression levels and subcellular localization. While nuclear p21 prevents cell cycle progression by inhibiting CDKs, cytoplasmic p21 exhibits anti‐apoptotic activity by inhibiting pro‐apoptotic proteins [27].

**Fig. 10:**
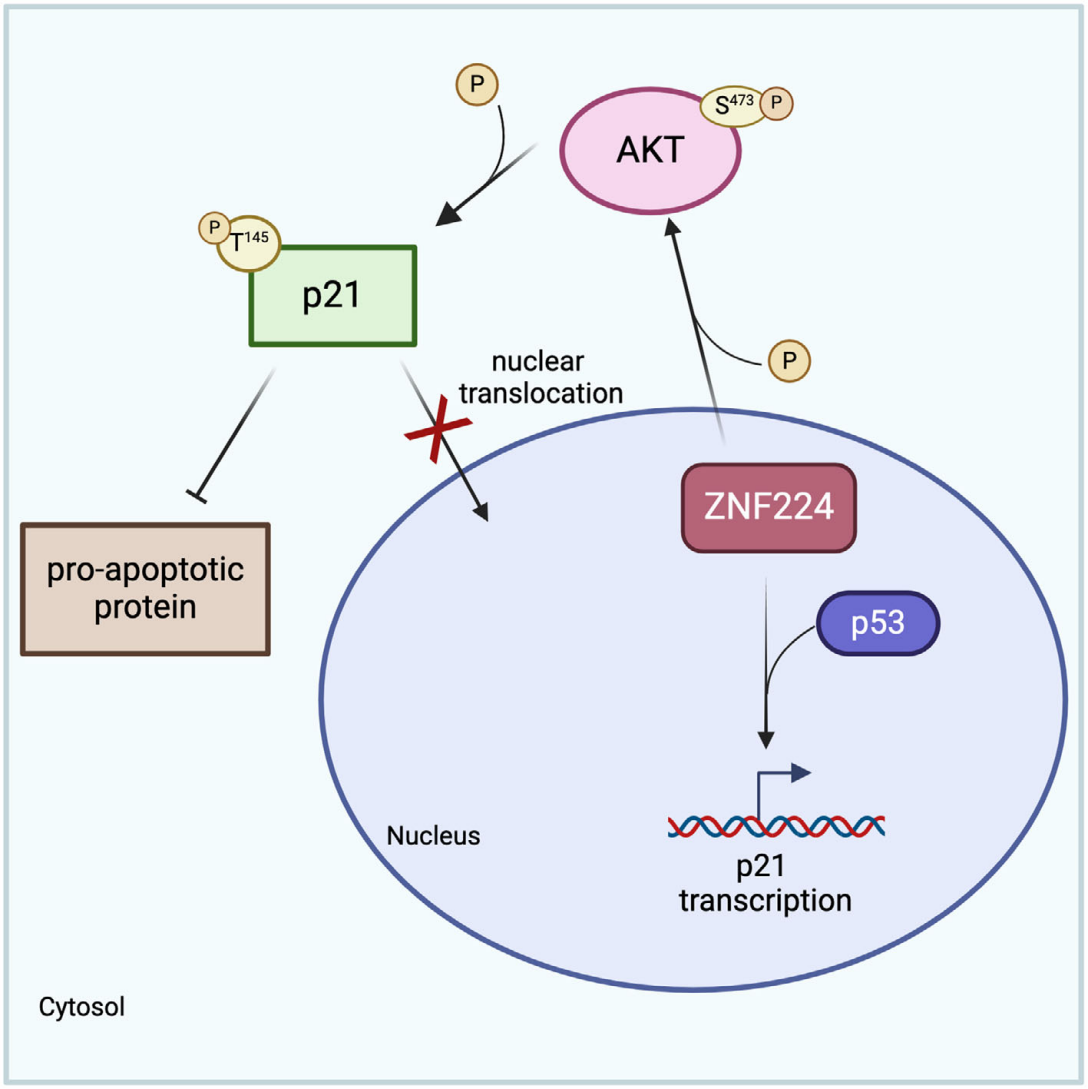
Graphical representation of ZNF224 pro‐oncogenic effects in melanoma cells by regulating p21 activity. In melanoma cells, ZNF224 overexpression promotes p21 protein cytosolic retention via activation of the AKT pathway, thus inducing the block of apoptosis and nuclear p21 cytostatic function. Moreover, ZNF224 regulates p21 expression levels at the transcriptional level via p53.

In this work, we found that ZNF224 overexpression enhanced AKT‐triggered p21 oncogenic effects, thereby inhibiting drug‐induced apoptosis and favoring cell growth. Furthermore, we show that ZNF224 positively modulated p21 gene transcription in a p53‐dependent manner (Fig. 10).

The role of p53 in joining ZNF224 to the p21 was also investigated in human melanoma collections of samples available in public repositories: through correlation analysis, the expression of p21 was initially found significantly positively correlated with ZNF224 in two of four analyzed groups of patients (Table 2). By applying an analysis procedure developed ad hoc to infer p53 effectiveness in regulating its target genes based on the bicor values of their expression levels (Fig. 6), we obtained evidence that ZNF224 and p21 expression levels are positively correlated in patients with no deducible alteration in p53 function, whereas, where p53 can be reasonably considered inactive, ZNF224/p21 correlation was lost. Removal of samples with impaired p53 activity allowed the restoration of the ZNF224/p21 correlation in the GSE7553 set but not in GSE15605, where, however, bicor values are not significant enough to be considered. Based on this evidence, the role of ZNF224 in transcriptionally activating p21 via p53, seen in melanoma cell lines, could also be at least plausible in melanoma samples from patients.

The induction of p21 expression by ZNF224 and its dependence on wild‐type p53 might appear paradoxical, for the increase of p21 mediated by p53 usually leads to cell cycle blockage and apoptosis. Numerous evidence prove that p21 plays oncogenic activities and is overexpressed in various human cancers, including prostate, breast, ovarian, and melanoma ones [23, 26, 27].

Based on the results of this work, a new role for ZNF224 can be conceived in A375 melanoma cells. Indeed, its overexpression causes resistance to apoptosis by hindering the induction of p53 and p21 by cisplatin and by increasing p21 phosphorylation via the activation of AKT, a kinase that has an important role in melanoma initiation and therapeutic resistance [43]. In A2058 cells, with impaired p53 DNA‐binding activity, it is possible to hypothesize that ZNF224 induces resistance to apoptosis through different molecular mechanisms.

Literature data indicate that in the AKT‐mediated survival pathway, p21 can be phosphorylated on Thr^145^ and/or Ser^146^, the relevant modifications for its subcellular distribution and stability. Phosphorylation of Thr^145^ inhibits p21 nuclear translocation, thus increasing its cytosolic accumulation associated with its pro‐oncogenic function, whereas phosphorylation on Ser^146^ significantly increases p21 protein stability [40].

Interestingly, in proliferating A375 cells, the ectopic expression of ZNF224 induces the cytosolic accumulation of p21 to the detriment of its nuclear share and pro‐apoptotic and cytostatic functions. Furthermore, in cisplatin‐treated cells, the overexpression of ZNF224 is accompanied by a decrease in p53 and p21 expression levels and in their cytosolic accumulation. Our results agree with literature data showing the role of cytoplasmic p21 in determining the resistance to conventional therapy based on molecules like cisplatin [37].

We partially elucidated the molecular mechanism responsible for the observed cytoplasmic distribution of p21 following ZNF224 overexpression. Indeed, our experiments demonstrated that ZNF224 may act as a regulatory component of the AKT/p21 pathway in A375 melanoma cells, being involved in the activation of AKT and p21 phosphorylation.

However, we observed that, also in cells lacking functional p53 (A2058), ZNF224 regulates proliferation via AKT activation. The role of p21 and its transcriptional downregulation by ZNF224 in conditions of impaired p53 transcriptional activity deserves further investigation to better characterize the role of ZNF224 in cancers with different p53/p21 assets, by studying its possible involvement in tumor progression in terms of growth and invasion potential [44, 45, 46]. It is possible to hypothesize that the observed down‐modulation of p21 by ZNF224 could lead to low expression levels of p21 relative to its physiological concentration, which then functions as a modulator of other oncogenic processes associated with the acquisition of aggressive tumor phenotype via p53‐independent mechanisms.

Taken together, we speculate that one way used by ZNF224 to define melanoma phenotype may be the concurrent control of the functional activity of wild‐type p53 and/or oncogenic AKT signaling pathway activation. Given the relevant role of ZNF224 in the NF‐kB pro‐survival pathway activation in some tumors [7, 15], we could hypothesize that it induces the pro‐survival activity of wild‐type p53 in melanoma cells, stimulating its capability to drive NF‐kB signaling activation and pro‐inflammatory cytokine production [47].

Therefore, the role of ZNF224 seems quite relevant in orchestrating multiple oncogenic events for tumor initiation and progression.

A better elucidation of the tumorigenic activities of ZNF224 still requires much effort. However, considering its role in the regulation of multiple proliferative and anti‐apoptotic signaling pathways, therapeutic strategies based on the down‐modulation of ZNF224 could constitute an attractive adjuvant in the treatment of melanoma.

## Materials and methods

### Cell cultures and treatments

A375 (RRID: CVCL_0132, as listed in the ExPASy Cellosaurus database) from human primary melanoma, A2058 (RRID: CVCL_1059, as listed in the ExPASy Cellosaurus database) from high metastatic melanoma, harboring *wild‐type* and mutated p53, respectively [48, 49].

(https://depmap.org/portal/cell_line/A2058_SKIN?tab=overview), HCT116 (RRID:CVCL_0291, as listed in the ExPASy Cellosaurus database) from human colon cancer p53 wild‐type (HCT116 p53+/+) and p53 null (HCT116 p53−/−) [50], were obtained from CEINGE Biotecnologie Avanzate (Naples, Italy), Cell Culture Facility (https://www.ceinge.unina.it/en/cell‐cultures). Cell lines were routinely tested for mycoplasma by using a Mycoplasma PCR detection kit (Applied Biological Materials, Vancouver, Canada).

All cells were cultured in Dulbecco's modified Eagle's medium (DMEM) (Corning, AZ, USA) supplemented with 10% fetal bovine serum (FBS) (Corning) and maintained at 37 °C in a humidified atmosphere with 5% CO_2_. For some experiments, A375 and A2058 cells were treated with 20 μm cisplatin (Accord Healthcare, Milan, Italy) or 20 μm etoposide (Teva Pharmaceutical Industries Ltd, UK) or 5 μm AKT inhibitor IV (Merck Millipore, MA, USA) for the indicated times.

### Transient transfections and dual‐luciferase reporter assays

For ZNF224 overexpression, A375 and A2058 cell lines were grown in subconfluency and transiently transfected with the expression vector p3x‐Flag ZNF224 or the empty vector p3x‐Flag as control, using Metafectene (Biontex, Munchen, Germany) according to the manufacturer's instructions.

To perform luciferase reporter assay, cells were cotransfected with increasing amounts (200, 400, 600 ng) of p3x‐FlagZNF224 or p3x‐Flag and 200 ng of a reporter plasmid containing a 2320 bp long fragment of the human p21 promoter region, cloned upstream of the luciferase reporter gene (promP21), or a reporter plasmid bearing a deletion in the p21 promoter region that includes the two p53 DNA‐binding sites (promp21/124) [34]. We also transfected cells with p53 H175 or p53 wt plasmids encoding for mutant or wild‐type p53 protein, respectively [35]. Cells were lysed 48 h after transfection and used for the Dual‐Luciferase assays (Dual‐Luciferase Reporter assay system, Promega, Madison, WI, USA) as previously described [6]. Dharmacon™ ON‐TARGET plus Human ZNF224 siRNA –SMART pool was used for ZNF224 knockdown in A375 and A2058 cells. A nontargeting pool was used as a scrambled negative control. Transfection of siRNAs was performed as previously described [20]. All experiments were performed at least three times.

### RNA extraction, reverse transcription, and real‐time q‐PCR

The extraction of total RNA from A375 and A2058 melanoma cell lines and HCT116 p53+/+ and HCT116 p53−/− colon cancer cell lines overexpressing or silenced for ZNF224 was carried out using the Quick‐RNA MiniPrep (ZymoResearch, Irvine, CA, USA), according to the protocol of the manufacturer.

Reverse transcription and quantitative real‐time PCR analysis were performed as previously described [51]. The mRNA amplification was performed on a CFX96 real‐time system (Bio‐Rad Laboratories, Hercules, CA, USA) using specific primers for ZNF224, HPRT, CCND3 [6], CCND1, BCL2 [7], MYC [12], and BAK1 [11], as previously reported. The housekeeping gene HPRT was used as an endogenous reference gene.

The sequences of other primers are: p21 Forward: CTGGAGACTCTCAGGGTCGAA; p21 Reverse: CGGCGTTTGGAGTGGTAGAA; PCNA Forward: CTGCAGAGCATGGACTCGTC, and PCNA Reverse: GTAGGTGTCGAAGCCCTCAGA.

### Protein extraction and western blot

As mentioned [52], whole‐cell protein extracts were produced from cell pellets using a modified RIPA buffer (Thermo Fisher Scientific, Waltham, MA, USA). According to the instructions provided by the manufacturer, NE‐PERTM Nuclear and Cytoplasmic Extraction Reagents (Thermo Scientific) were used to prepare nuclear and cytoplasmic extracts.

Following the manufacturer's directions, the protein extracts were resolved on SDS/PAGE and transferred to a nitrocellulose membrane using an RTA Transfer Kit (Bio‐Rad) and Trans‐Blot Turbo (Bio‐Rad). The following antibodies were used in western blot: Anti‐FLAG (Sigma‐Aldrich, Milan, Italy, dilution 1:1000); anti‐ZNF224 (rabbit polyclonal, T3) [20], anti‐Myc (Aviva System Biology, CA, USA, dilution 1:500) anti‐BCL2 (Santa Cruz Biotechnology, CA, USA, dilution 1: 500); anti‐p53 (Santa Cruz Biotechnology, dilution 1:1000); p21 (Cell Signaling, Danvers, MA, USA, dilution 1:2000); β‐actin (Cell Signaling, dilution 1: 1000); β‐Tubulin (Merck Millipore, dilution 1:1000); Lamin A/C (Cell Signaling, dilution 1:1000); LDH (Santa Cruz Biotechnology, dilution 1:1000); AKT (Santa Cruz Biotechnology, dilution 1:500); p‐AKT Ser^473^ (Cell Signaling, dilution 1:1000); p‐p21 Thr^145^ (GeneTex, CA, USA, dilution 1:5000). The experiments were performed at least three times and western blots shown in the figures are representatives from one of these experiments. Densitometric analysis was performed by imagej software.

### Proliferation assay

The proliferation analysis of melanoma cells was performed using The CellTrace™ CFSE Cell Proliferation Kit (Thermo Fisher Scientific). A375 and A2058 cells were harvested 24 h after transfection and stained for 10’ at room temperature with 400 nm CellTrace™ CFSE. Staining was stopped by washing the cells with a complete medium and keeping them on ice for 5 min. After that, the cells were plated in six‐well plates in triplicate at 2 × 10^5^/well for a further 24 and 48 h, then harvested, washed with PBS 1×, incubated for 10’ with 7‐AAD viability labeling (Invitrogen, CA, USA) to exclude non‐viability cells, and finally analyzed using BD Accuri™ C6 Cytometer (Becton, Dickinson and Company BD). The flow cytometry data were analyzed by C6 Accurì software. The obtained CFSE fluorescence decay is proportional to the increase in proliferation rate. In the histogram, the percentage of proliferation has been reported for all data points. Experiments were performed twice in triplicate.

### Caspase activity assay

A375 and A2058 cells, 24 h after transfection of the ZNF224 overexpression plasmid or siRNA, were treated with 20 μm cisplatin (Accord Healthcare) or 20 μm etoposide (Teva) for 16 h. Cells were assayed for apoptosis using The Caspase‐Glo® 3/7 Assay (Promega) according to the manufacturer's instructions. For all data points, the luminescent signals in response to caspase‐3/7 activities are reported. Experiments were performed three times in triplicate.

### Statistical analysis of expression data

Data were assessed as the mean ± standard deviation (SD) of at least three separate experiments or two duplicates. GraphPad Prism 7 (GraphPad Software, Inc., San Diego, CA, USA) was used for graphs and data analysis. Statistical significances were determined through the *t*‐test analysis or ANOVA test comparing results between the control and test cells. Differences were statistically significant when *P* ≤ 0.05 (*) or highly significant when *P* ≤ 0.01 (**).

### Analysis of transcriptomic datasets including melanoma samples

Transcriptomic datasets GSE46517 [31], GSE19234 [32], GSE7553 [33], and GSE15605 [53] from human melanoma patients transcriptomic profiling experiments were obtained from the public database Gene Expression Omnibus (GEO) at NCBI (National Centre For Biotechnology Information, https://www.ncbi.nlm.nih.gov/geo/) and analyzed within the R environment (http://www.r‐project.org). Raw data were preprocessed by using the Robust Multi‐Array Average (RMA) method available within the affy package (https://www.rdocumentation.org/packages/affy/versions/1.50.0) to obtain the background‐corrected, normalized, log‐transformed probe intensities. Bi‐weight mid‐correlation (bicor) was calculated using the WGCNA package [55]. The procedure used for inferring p53 effectiveness in regulating the expression level of its target genes uses the nls (nonlinear least squared) function for curve fitting analyses and was also implemented and run within the R environment.

## Conflicts of interest

The authors declare no conflict of interest.

## Author contributions

LS developed and applied bioinformatic procedures for analyzing public datasets and wrote the manuscript. UC designed and performed *in vitro* studies and analyzed the data. DSdV performed *in vitro* experiments, ET analyzed the public datasets and performed statistical analysis. MT performed *in vitro* experiments. GS revised the manuscript. RR performed *in vitro* experiments. SR helped to analyze and interpret the flow cytometric results. MG revised the manuscript. GP contributed to developing the bioinformatic procedures, analyzing the bioinformatic data, and revising the manuscript. AL interpreted the data and wrote the manuscript. PC designed and coordinated the study and wrote the manuscript. EC conceived and designed the study, performed the experiments, and wrote the manuscript. All authors have read and approved the final manuscript.

## Supporting information


**Fig. S1.** Four category distribution of p53/target genes correlation levels reported as the stacked‐bar representation.
**Table S1.** Expression correlation between p53 and 59 p53 transcriptional target genes.

## Data Availability

The data supporting the findings of this study are openly available in the Gene Expression Omnibus database at NCBI (https://www.ncbi.nlm.nih.gov/geo/) under accession numbers [GSE46517, GSE19234, GSE15605, GSE7553].
