# Supplementary material for: ZNF224 enhances the oncogenic function of p21 via p53 and AKT pathways in melanoma
Source: FEBS J. 2025 May 5;292(15):3986–4005. doi: 10.1111/febs.70114 (PMC12326943; doi:10.1111/febs.70114)
Supplement: Supplementary file 1 — Fig. S1. Four category distribution of p53/target genes correlation levels reported as the stacked‐bar representation. Table S1. Expression correlation between p53 and 59 p53 transcriptional target genes. [file FEBS-292-3986-s001.pdf]

Table S1: Expression correlation between p53 and 59 p53 transcriptional target genes

	GSE46517		GSE15605			GSE7553		
	Bicor	P value	Bicor	p value	< ref	Bicor	p value	< ref
RABGGTA	0.80	1.2E-19	0.54	1.0E-05	1	0.66	3.3E-08	1
PTEN	0.79	1.1E-18	0.53	1.6E-05	1	0.49	1.1E-04	1
TSC2	0.78	2.3E-18	0.66	1.4E-08	1	0.79	7.7E-13	0
PML	0.78	6.1E-18	0.50	6.9E-05	1	0.76	1.3E-11	1
PLK3	0.78	7.5E-18	0.68	5.4E-09	1	0.62	3.0E-07	1
PRKAB1	0.75	6.4E-16	0.73	5.4E-11	1	0.76	1.1E-11	0
HTT	0.74	7.2E-16	0.55	7.5E-06	1	0.62	2.9E-07	1
DDB2	0.73	5.9E-15	0.52	2.5E-05	1	0.41	1.5E-03	1
APC	0.73	6.3E-15	0.64	7.6E-08	1	0.69	3.5E-09	1
BTG2	0.73	7.5E-15	0.61	3.5E-07	1	0.54	1.9E-05	1
MDM2	0.72	1.2E-14	0.77	2.4E-12	0	0.44	6.3E-04	1
TRPM2	0.72	2.4E-14	0.70	1.3E-09	1	0.64	1.2E-07	1
BCL6	0.71	3.7E-14	0.50	5.4E-05	1	0.65	4.9E-08	1
ARID3A	0.71	4.0E-14	0.73	5.4E-11	0	0.67	1.6E-08	1
EEF1A1	0.71	4.1E-14	0.67	8.4E-09	1	0.57	4.0E-06	1
FAS	0.71	8.3E-14	0.37	4.7E-03	1	0.34	1.1E-02	1
CCNK	0.70	1.1E-13	0.69	1.9E-09	1	0.64	9.1E-08	1
HGF	0.70	1.4E-13	0.48	1.2E-04	1	0.49	1.4E-04	1
CASP6	0.70	1.4E-13	0.79	1.2E-13	0	0.62	3.4E-07	1
CD82	0.70	1.8E-13	0.38	3.5E-03	1	0.61	6.4E-07	1
P2RX6	0.70	2.0E-13	0.74	2.3E-11	0	0.68	7.7E-09	1
TNFRSF10D	0.69	3.6E-13	0.23	7.6E-02	1	0.46	3.8E-04	1
RB1	0.69	4.1E-13	0.72	1.8E-10	0	0.61	7.5E-07	1
CDKN1A	0.69	5.2E-13	0.46	3.0E-04	1	0.35	8.4E-03	1
PRDM1	0.69	5.3E-13	0.42	9.4E-04	1	0.41	1.7E-03	1
MLH1	0.68	1.8E-12	0.55	8.7E-06	1	0.67	1.2E-08	1
TP73	0.67	4.1E-12	0.69	2.8E-09	0	0.65	4.6E-08	1
TRIAP1	0.67	5.0E-12	0.61	2.8E-07	1	0.43	8.4E-04	1
GPX1	0.67	6.2E-12	0.61	3.4E-07	1	0.43	8.9E-04	1
BBC3	0.66	1.4E-11	0.68	4.7E-09	0	0.60	1.0E-06	1
HIC1	0.66	1.5E-11	0.67	8.8E-09	0	0.63	2.4E-07	1

**FANCC**	0.65	2.0E-11	0.67	9.2E-09	0	0.68	5.9E-09	0
**CTSD**	0.65	2.0E-11	0.61	3.3E-07	1	0.40	2.6E-03	1
**TGFA**	0.65	2.4E-11	0.43	6.8E-04	1	0.72	3.4E-10	0
**EGFR**	0.65	2.5E-11	0.26	5.2E-02	1	0.62	4.1E-07	1
**BNIP3L**	0.65	4.0E-11	0.41	1.3E-03	1	0.43	9.4E-04	1
**TP63**	0.65	4.2E-11	0.21	1.1E-01	1	0.59	2.1E-06	1
**MET**	0.64	4.7E-11	0.43	7.9E-04	1	0.26	4.8E-02	1
**VDR**	0.64	4.7E-11	0.47	2.1E-04	1	0.38	3.8E-03	1
**STEAP3**	0.63	1.6E-10	0.49	9.1E-05	1	0.56	6.7E-06	1
**ATF3**	0.63	1.9E-10	0.60	7.9E-07	1	0.60	1.0E-06	1
**TNFRSF10C**	0.62	3.3E-10	0.72	1.3E-10	0	0.58	2.9E-06	1
**SCN3B**	0.61	7.2E-10	0.22	9.1E-02	1	0.67	2.0E-08	0
**DDIT4**	0.57	1.4E-08	0.52	2.8E-05	1	0.68	6.3E-09	0
**DUSP5**	0.57	2.2E-08	0.47	1.9E-04	1	0.56	6.3E-06	1
**COL18A1**	0.56	4.1E-08	0.53	1.8E-05	1	0.53	2.2E-05	1
**GADD45A**	0.56	5.1E-08	0.48	1.5E-04	1	0.43	1.0E-03	1
**HRAS**	0.54	1.1E-07	0.35	6.4E-03	1	0.40	2.2E-03	1
**BDKRB2**	0.52	3.6E-07	0.19	1.5E-01	1	0.48	1.7E-04	1
**DUSP1**	0.52	4.0E-07	0.62	2.0E-07	0	0.63	2.5E-07	0
**RGCC**	0.52	6.3E-07	0.43	8.3E-04	1	0.48	2.0E-04	1
**PCBP4**	0.50	1.4E-06	0.58	1.7E-06	0	0.71	9.8E-10	0
**BAI1**	0.50	1.4E-06	0.65	2.5E-08	0	0.56	6.8E-06	0
**VCAN**	0.48	3.8E-06	0.49	8.3E-05	0	0.44	6.2E-04	1
**IGFBP3**	0.43	5.3E-05	0.62	1.7E-07	0	0.57	3.9E-06	0
**PYCARD**	0.40	1.6E-04	0.36	4.9E-03	1	0.13	3.4E-01	1
**NDRG1**	0.30	5.1E-03	0.30	2.0E-02	1	0.54	1.6E-05	0
**PERP**	0.13	2.3E-01	0.10	4.7E-01	1	0.07	6.3E-01	1
**S100A2**	-0.03	7.9E-01	0.04	7.8E-01	0	0.01	9.3E-01	0
					43			47

The correlation between p53 and a panel of 59 p53 transcriptional target genes from Riley et al. (36) was tested in GSE15605 and GSE7553 and compared with GSE46517, used as a reference: the results are reported in the table, where cells containing bicor values were filled according to the following rules: above 0.7, dark green; between 0.5 and 0.7, light green; below 0.5, yellow; below 0.3, red. Bicor values calculated for GSE15605 and GSE7553 show lower values than GSE46517 in 43 and 47 cases out of 59

Fig. S1: Four category distribution of p53/target genes correlation levels reported as stacked-bar representation.

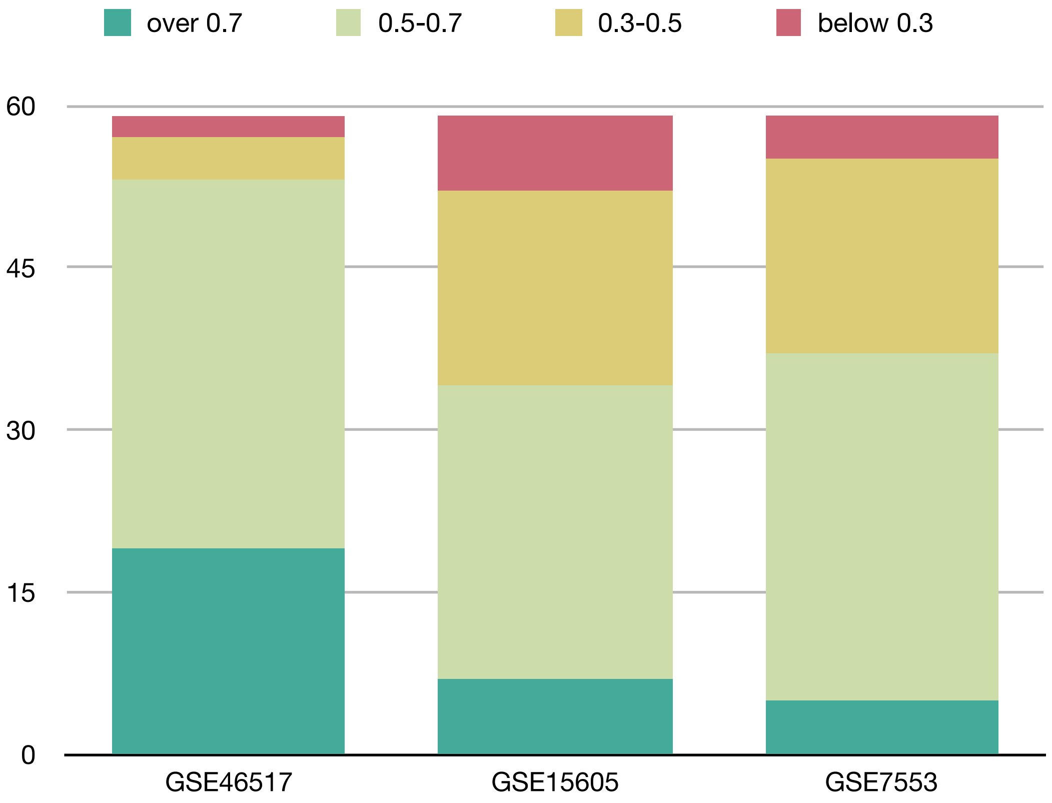

Using the same ranges, the fraction of target genes whose correlation with p53 falling in each category was calculated. The stacked-bar graph shows that in GSE15605 and GSE7553 correlation values are worse than in the reference dataset (GSE46517).
